# Pre-pregnancy community-based intervention for couples in Malaysia: application of intervention mapping

**DOI:** 10.1186/s12889-016-3827-x

**Published:** 2016-11-17

**Authors:** Shane A. Norris, Julius Cheah Chee Ho, Aswir Abd Rashed, Vibeke Vinding, Jutta K. H. Skau, Regien Biesma, Jens Aagaard-Hansen, Mark Hanson, Priya Matzen

**Affiliations:** 1 MRC Developmental Pathways for Health Research Unit, Department of Paediatrics, School of Clinical Medicine, Faculty of Health Sciences, University of the Witwatersrand, Johannesburg, South Africa; 2 School of Medicine and Health Sciences, Monash University Malaysia, Jalan Lagoon Selatan, 46150 Bandar Sunway, Selangor Malaysia; 3 Nutrition Unit, Cardiovascular, Diabetes and Nutrition Research Center, Institute for Medical Research, Ministry of Health Malaysia, Jalan Pahang, 50588 Kuala Lumpur Malaysia; 4 IT University of Copenhagen, Rued Langgaards Vej 7, 2300 Copenhagen, Denmark; 5 Department of Epidemiology and Public Health Medicine, Royal College of Surgeons in Ireland, 123 Saint Stephen’s Green, Dublin 2, Ireland; 6 Human Development and Health, Faculty of Medicine, University of Southampton, Southampton, UK; 7 Health Promotion Research, Steno Diabetes Center, Niels Steensens Vej 6, 2820 Gentofte, Copenhagen Denmark; 8 Institute of Developmental Sciences, British Heart Foundation, Cardiovascular Sciences Faculty of Medicine, University of Southampton Mailpoint 887, Southampton General Hospital Tremona Road, Southampton, SO16 6YD UK

**Keywords:** Metabolic disease risk, Intervention mapping, Malaysia, Reproductive health, Gestational diabetes mellitus

## Abstract

**Background:**

Malaysia is experiencing a nutrition transition with burgeoning obesity, particularly in women, and a growing prevalence of non-communicable disease. These health burdens have severe implications not only for adult health but also across generations. Pre-conception health promotion could address the intergenerational risk of metabolic disease. This paper describes the development of the “Jom Mama” intervention using Intervention Mapping (IM). The Jom Mama intervention aims to improve the health of young adult couples in Malaysia prior to conception.

**Methods:**

IM comprises of five steps prior to the last one, which involves the evaluation of the intervention. We used the five steps to develop the Jom Mama intervention.

**Results:**

Both the process and evidence is documented providing the rationale to the selection of the key objectives of the intervention: (i) increasing healthy dietary practice; (ii) increasing physical activity levels, (iii) reducing sedentary activity; and (iv) improving social support to offset stressful lifestyles. From the IM process, Jom Mama will be health-system centred approach that uniquely combines both community health promoters and an electronic-health platform to deliver the complex intervention.

**Conclusion:**

IM is an iterative process that systematically gathers “best” evidence, selects appropriate theories of behaviour change, and facilitates formative research so as to develop a complex intervention. Though the IM process is time consuming, complex, and costly, it has enriched the Jom Mama intervention with a number of notable advantages: (i) intervention fashioned on formative work with stakeholders and in the target group; (ii) intervention combines research evidence with theory; (iii) intervention acknowledges multiple dynamics of influence; and (iv) intervention is embedded within health service priorities in Malaysia for greater scale-up possibility.

## Background

Non-communicable disease (NCD) is anticipated to be responsible for 73 % of all deaths worldwide by 2020 [1]. Malaysia, like many middle-income countries, is undergoing a nutrition transition coupled with rapid urbanisation leading to shifts in physical and sedentary activity, and dietary practices. Within a decade (1996–2006) in Malaysia there was an astounding 250 % rise in obesity, 88 % upsurge in type-2 diabetes (T2D), and 43 % rise in hypertension [2]. The National Health Morbidity Survey of 2011 [2] demonstrated that the prevalence of T2D among young adults (18-34 years) spanned between 2.1 % and 9.4 %, and incidence of hypertension, dyslipideamia, and central adiposity ranged between 8.1 and 22.2 %, 11.3–30.4 %, and 19.6–44.7 % respectively. Reducing the metabolic disease risk profile in young adults may be a critical strategy to combatting the NCD burden in Malaysia. In turn, optimising pre-pregnancy health in young women may also provide a unique window of opportunity to reduce metabolic disease risk intergenerationally, as pre-pregnancy obesity has been associated with greater risk for gestational diabetes, poorer birth outcomes and greater risk for obesity and metabolic disease in the offspring [3].

Designing and evaluating complex health service interventions targeting women pre-pregnancy, obtaining support from stakeholders and local communities, recruiting participants and maintaining high retention rates, and ensuring integration and scale-up into existing health services is not an easy task in any population [4]. Guidelines and theoretical frameworks are useful tools to support the development, design and evaluation of such complex interventions. Intervention Mapping (IM) is one of such framework that has been described as a advantageous approach for the development of complex interventions [5–10]. The benefits of the IM approach include: (i) its capacity to take into account the complexity of intervention development and research; and (ii) grounding interventions in theory and evidence [8].

In Malaysia, pre-pregnancy care exists as part of maternal and child health services that comprises of pre-marital HIV screening and a general wellness programme. The main aim of this health service is to provide men and women of reproductive age with an avenue to achieve a safe and successful pregnancy. In particular, the services include: (i) Screening for risk factors and counsel future mothers to reduce maternal and prenatal morbidity and mortality; (ii) Enabling prospective parents and women of reproductive age to plan for a healthy pregnancy through provision of health education; and (iii) Emphasising the importance of healthy living. This foundation of pre-pregnancy care could be strengthened to also tackle cardiometabolic disease risk in younger adults. However, often government departments have the desire to develop and evaluate complex interventions but do not have any experience with tools that could assist with this process. Therefore, the aim of this paper was to apply and discuss IM in the development of a government pre-pregnancy community-based intervention to improve the health of married couples in Malaysia.

## Method

### Setting and population

The Jom Mama Project, which means ‘come on mom’ in Malay, started in 2011 as a partnership between the Ministry of Health of Malaysia and collaborating institutions. The overarching hypothesis of Jom Mama is that maternal pre-conception adiposity, weight gain during pregnancy, and hyperglycaemia, can impact on pregnancy complications (for example, obstructive labour), maternal postnatal risk of T2D, and offspring risk for obesity and T2D. Capitalising on the existing pre-pregnancy health service infrastructure in Malaysia, we hypothesised that by strengthening the health system with greater emphasis on pre-pregnancy health through a combination of community health promoters (CHPs) and an electronic-health system (web-based platform), we could improve women’s health prior to pregnancy and that this would have benefits for both women and their offspring. The city of Seremban within the state of Negeri Sembilan, which is situated 70 km from Kuala Lumpur (Malaysia), was selected as the study site. Seremban has one of the highest prevalence of T2D in Malaysia at 22 %, compared to the national average of 15.2 % [2] and the city’s population profile reflects the national demographic composition.

### Intervention mapping process

IM involves six steps: (i) Needs assessment; (ii) Formulation of change objectives (intervention objectives); (iii) Selection of theory-based approaches; (iv) Intervention development; (v) Development of adoption and implementation plan; and (vi) Evaluation. We systematically applied these steps to develop a pre-pregnancy complex intervention.

## Results

### IM Step 1: Needs assessment

Step 1 involved the identification of “health needs” and associated factors. To complete this step of the IM process, three study components were developed and implemented: (i) A stakeholder engagement and consensus study; (ii) Formative qualitative research; and (iii) A literature review.

#### Stakeholder engagement and consensus

The process of identifying the “health needs” or “health burden” included both a top-down (national policy-makers and community stakeholders) and a bottom-up (target intervention group of young adults) approach. Stakeholder engagement comprised of two components: (i) Achieving consensus amongst a group of policy makers, stakeholders, and target intervention participants (young adults) on the critical factor/s impacting the health of young adults in Malaysia, and (ii) Formulating a cardiometabolic disease prevention framework. A Knowledge Resource Nomination Worksheet was drawn-up to indicate which types of stakeholders were needed to provide insights into young adult health in Malaysia. From the invitations sent out to senior members of 19 Malaysian ministries and young adults we recruited 33 key stakeholders. From these stakeholders, using the Delphi technique, we achieved consensus through 3 iterative rounds that the high cost of living (life stress) was the most significant challenge young adults in Malaysia face [11].

Stakeholders were invited to participate in a workshop following the Delphi consensus process to discuss the results, and participate with scientists to realise a shared understanding of the most important issue/s young adults face and how it may exacerbate poor health (obesity and T2D). From the engagement exercise, it emerged that the difficulty with choosing or continuing a healthy lifestyle was primarily disrupted by life stress. The high cost of living was seen as the main impetus why younger working adults are propelled to attain better financial security, and in turn this leads to a less focus on health. The conceptualised metabolic disease prevention framework proposed by the stakeholders prioritised the need to assist young adults to cope with daily life stress so as to make health a priority by eating healthier and being more physically active.

#### Qualitative formative research

Three extensive qualitative studies were completed in the study community of Seremban as part of formative research [12]. Through purposive sampling to ensure maximum variation, the following categories of informants were interviewed: 21 community leaders (local politicians, religious leaders, public health experts, private business persons and representatives from civil society), 12 healthcare service providers (doctors and nurses) from 2 healthcare centres in Seremban, 18 young couples (pre-pregnancy state) and nine related influencers (individuals who may have a significant influence on the lifestyles of young couples for example mothers, grandmothers, peers). Semi-structured qualitative interviews were conducted based on question guides, which had been piloted and adapted prior to interviews.

In summary from the formative work, the community leaders identified several barriers for couples to practice healthier lifestyles, these included: (i) Financial constraints and stress; (ii) Demanding commuting and working life with less time to prioritise healthy living; (iii) Lack of knowledge, awareness, and health literacy, (iv) Built environment (for example heavy traffic and poor pedestrian walkways and shortage of suitable indoor sports and recreational facilities) and (v) Socio-cultural factors (for example the high consumption of coconut milk, palm oil and sugar in food preparation). The healthcare provider interviews noted that the HIV and maternal and child health programmes in the community clinics are already established and these programmes may have the potential to be strengthened optimise pre-pregnancy health [12].

The young adults (couples) articulated that excessive consumption of sugar and lack of physical activity was the main causes of cardiometabolic disease, in particular T2D. Despite the fact that they were aware of the connection between the mother’s pre-conception health and the health of the baby, their work pattern (long working hours, irregular shift hours) and lifestyle resulted in poorer eating habits and less engagement in physical activity. The influential factors on dietary patterns were the convenient access of less healthy foods and limited availability of healthy food options particularly in the working environment. Young couples obtained their health information from various sources including electronic and printed media, as well as from healthcare providers. Having a healthy baby was articulated as an important motivational factor to lead a healthier lifestyle. However, young couples expressed a preference for incentives (for example free vouchers) to motivate them to stay on any intervention programme and coupled with strong spousal support. The content of the programme should be simple, attractive, and not interfere with their working hours. Also, they strongly suggested that it would be preferable for these services to take place outside of the clinic [12].

#### Literature reviews

To supplement the formative work, we systematically reviewed literature in Malaysia on T2D and its underlying determinants [13]. In Malaysia, the main socio-demographic determinants of adult obesity and T2D were: (i) being female; (ii) of Malay and Indian ethnicity; (iii) and less education. Furthermore, increased fast food intake, greater alcohol and tobacco use, and reduced exercise and increased sedentary activity were also associated with obesity and T2D risk [13]. We reviewed evidence-based guidelines and systematic reviews pertaining to diabetes prevention programmes. Such guidelines [14–16] typically focused on changing physical activity and dietary behavior. A systematic review and meta-analysis found that modest lifestyle interventions are effectual at weight loss and some risk reduction of T2D [17].

### IM Step 2: formulation of change objectives

An important part of the IM process is to cascade the evidence generated in step 1 into identifying and mapping out key behavioural outcomes that will be incorporated into the intervention. The key behavioural outcomes selected for the Jom Mama intervention based on the evidence were: (i) Choosing healthier dietary choices more often; (ii) More exercise; (iii) Less sedentary activity; and (iv) Greater social support to better cope with stressful lifestyles. These behavioural aims are reflected as change objectives in Table 1.

**Table 1:** Behavioral outcomes and change objectives

	Behavioural outcomes to reduce metabolic disease risk			
	Healthy balanced diet	Increase physical activity	Reduce sedentary behaviour	Reduce stress
Change objectives	Increase consumption of fruits and vegetables Reduce portion size of starchy staple foods Decrease sugar consumption Decrease consumption of high fat foods Achieve the recommended levels of micronutrient intake	Achieving a minimum level of 30 mins of moderate to vigorous exercise per day to enhance fitness	Break up sitting time	Improve time management skills

Table describing the behavioural outcomes identified and the change objectives targeted for the Jom Mama intervention

### IM Step 3: selection of theory-based approaches

**Fig. 1:**
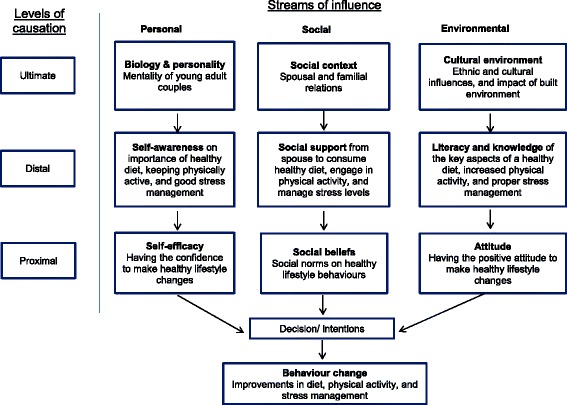
The Theory of Triadic Influence and the Jom Mama trial (adapted from Flay et al. 2009). Diagram describing the usage of the Theory of Triadic Influence in the Jom Mama intervention

In step 3, theory-based approaches were identified that could be suitable for effective behaviour change in young adults. We examined behaviour change techniques (BCTs) by reviewing the taxonomy developed by Abraham and Michie [18] and the systematic review by Flay et al. [19]. We adopted the Theory of Triadic Influence (TTI), which incorporates sociological and psychological understanding of behaviour and frames it into streams of influence (Fig. 1) [20–22]. The advantage of TTI is that it combines three domains and unifies both proximal and distal influences on behavior. In the Jom Mama study, we considered intra-personal proximal influences such as couples’ health awareness and attitudes, inter-personal influences such as health information and lifestyle factors, and distal socio-cultural influences such as the cultural setting of the study.

### Step 4: intervention development

#### Jom Mama intervention

A small working group (SAN, JCH, VV, AR, JAH) developed an intervention plan to achieve the behaviour change objectives and together with the results of the previous IM steps presented the plan to the Ministry of Health and all collaborators in August 2013. These presentations were then modified following the discussion and then presented to community representatives, religious leaders non-governmental organisations, and representatives of the health services in October 2013 for comment and input. The feedback from the stakeholders recognised the need and importance of the intervention and “supported” the intervention implementation and evaluation. This was then further discussed with representatives of various departments of the Ministry of Health for final modifications and approval (April 2014).

**Fig. 2:**
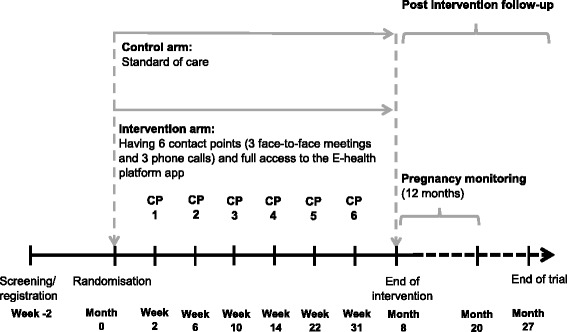
Jom Mama trial design. Overview of the trial design of the Jom Mama randomised control trial

Following sign off, the Jom Mama intervention was seen as a complex intervention embedded within the health system and aimed at young married couples with CHPs as change agents through the use of an E-health platform. A randomised-control trial design was selected to evaluate if a complex intervention combining motivational interviewing by trained CHPs and utilisation of a mobile E-health application to support the CHPs and participants would minimise abdominal obesity and diabetes risk in women prior to pregnancy through healthier diets, increased physical activity, reduced sedentary behaviour and reduce stress. The trial will enrol 264 pre-pregnant women and their spouses. Those in the intervention arm will be exposed to an 8-month intervention with 6 contact points with the CHP (Fig. 2 and Table 2). In addition, the intervention group will have access to a mobile application to promote a healthy lifestyle. The control group will not receive any specific intervention apart from the standard care currently provided in the existing health services. The trial is powered on waist circumference as the primary outcome. Secondary outcomes include differences in HbA1c, lipid profile, blood pressure, weight, hip circumference, waist-to-hip ratio, waist-to-height ratio, dietary patterns, physical activity, reported stress levels and health literacy [23].

**Table 2:** Overview of CHP contact point meetings

Contact point	Aims
Contact point 1 (Face-to-face)	Assessment of couples’ health risk and behaviour: risk assessment Introduce to use of mobile application Initiate review of assessments and behaviour change process Goal setting Creation of a Whatsapp mobile app chat group
Contact point 2 (Face-to-face)	Follow up on use of mobile application Provide health information as required Review and address extrinsic supportive and inhibiting factors Introduce proactive coping
Contact point 3 (Phone)	Follow up on use of mobile application Follow-up on progress based on initial goal setting Provide support and advice Provide health information as required Revise goal setting and set new goals as relevant
Contact point 4 (Phone)	Follow up on use of mobile application Follow up on progress based on previously set goals Provide support and advice Provide health information as required Revise goal setting and set new goals as relevant
Contact point 5 (Face-to-face)	Follow up on use of mobile application Follow up on progress based on previously set goals Provide support and advice Provide health information as required Revise goal setting and set new goals as relevant
Contact point 6 (Phone)	Follow up on progress based on previously set goals Provide support and advice Provide health information as required Goal setting and planning for the future End of intervention process

Details of the purpose of each contact point meeting between CHP and young couples in the Jom Mama trial

### IM Step 5: feasibility testing

Feasibility testing of the trial was conducted from February to August 2015 with the objective to assess the different processes of each phase of the trial and to obtain CHP and participant perceptions of the intervention through observation, focus group discussions, and semi-structured interviews. Participant recruitment was found to be a significant challenge, as the study aims to recruit relatively young and healthy individuals who may not have immediate health priorities. Furthermore, long and irregular working hours was also found to be a barrier for the young couples to commit to the intervention. The feasibility testing indicated that a face-to-face approach during pre-marital health screenings at primary health clinics to be the most optimal recruitment channel as compared to telephone calls and social media interaction. The intervention package was well-received by the participating young couples with particular appreciation for the engagement and support offered by the CHP. A challenge that emerged was that the usage of the Jom Mama E-health application was less than anticipated and limited due to poor internet connectivity in the Seremban district. A number of mitigating strategies have been put in place to meet the challenges of recruitment and lack of internet connectivity, such as a wider campaign to promote the project, and implementing Wifi hotspots in study clinics to enable participating couples to download the Jom Mama application.

A further component of the feasibility testing was the development of a training curriculum for the CHPs. The CHPs are public primary healthcare nurses employed in the Seremban district. They were recruited into the project on a voluntary basis based on their interest to be trained in motivational interviewing and E-health. The CHPs in the feasibility testing underwent a thorough five-day training session that focused on building skills in motivational interviewing and basic knowledge in nutrition and physical activity. In addition, the training also included a module on the E-health platform that consisted of a mobile application for couples, and a back-end interface for CHPs to monitor their engagement activities. The feasibility testing concluded that the trial was feasible with regard to recruiting young couples, the successful training of CHPs, intervention implementation, and good acceptability of the intervention material and process by both CHPs and couples.

### IM Step 6: evaluation

Working through IM steps 1–5 we completed the intervention development, and a trial evaluation protocol was formulated. The trial seeks to enroll Malaysian women (20–39 years) who are nulliparous not diagnosed with T2D and possess a smartphone with their spouses. These participants will be randomised to either the intervention or the control arm for an 8-month period. The primary outcome is change in waist circumference before (baseline) and after (endline) assessments. Secondary outcomes include: changes in body mass index, metabolic measures (glucose, lipid profile), and change in health literacy, dietary practices, physical activity, sedentary behaviour, and stress. The Medical Research and Ethics Committee of the Ministry of Health Malaysia (protocol number: NMRR-14-904-21963) has approved this trial. The trial commenced in 2015, and will be completed by 2017.

## Discussion

By applying the IM approach we can more confidently claim that the Jom Mama intervention is: (i) based on extensive formative work, stakeholder engagement, and “best practice” evidence from evaluated interventions; (ii) combines theory with scientific evidence to inform the intervention objectives; and (iii) embedded within Malaysian health system priorities for greater scale-up possibility. This is a key advantage of IM. However, IM process can be time consuming (3 years in our case), tiresome, complex, and requires funding, which may not always be readily available for intervention development. However, the advantages seem to outweigh the disadvantages in that the intervention development is more robust particularly as the intervention deals with the complex aim of changing behavior. The Jom Mama intervention is novel in that: (i) it deploys multi-prong prevention strategy prior to conception to optimise health that could not only offset cardiometabolic risk in the women but also in future offspring; (ii) pragmatic; and (iii) draws upon and enhances existing health service infrastructure and resources to ensure that if shown to be effective is more likely to be scalable..

## Conclusions

In conclusion the IM technique is a useful approach for ensuring that scientific evidence and theory together inform the development of the intervention components. The application of the IM approach also assisted in developing capacity within the Ministry of Health to engage with a tool that provides a more thorough iterative process to the development of a complex intervention that involves behaviour change.

## Abbreviations

BCT: Behaviour change technique
CHP: Community health promoters
IM: Intervention mapping
NCD: Non-communicable disease
T2D: Type 2 diabetes
TTI: Theory of triadic influence
